# Supplementary material for: Unveiling Liquid-Crystalline Lipids in the Urothelial Membrane through Cryo-EM
Source: bioRxiv. 2023 May 29:2023.05.29.542358. Preprint. [Version 1] doi: 10.1101/2023.05.29.542358 (PMC10312457; doi:10.1101/2023.05.29.542358)
Supplement: Supplementary file 1 [file NIHPP2023.05.29.542358V1-supplement-1.pdf]

bioRxiv preprint doi: https://doi.org/10.1101/2023.05.29.542358; this version posted May 29, 2023. The copyright holder for this preprint (which was not certified by peer review) is the author/funder, who has granted bioRxiv a license to display the preprint in perpetuity. It is made available under aCC-BY 4.0 International license.

Supplemental Materials

Supplemental Figures 1-3

Supplemental Table 1

Supplemental Spread sheet 1

bioRxiv preprint doi: https://doi.org/10.1101/2023.05.29.542358; this version posted May 29, 2023. The copyright holder for this preprint (which was not certified by peer review) is the author/funder, who has granted bioRxiv a license to display the preprint in perpetuity. It is made available under aCC-BY 4.0 International license.

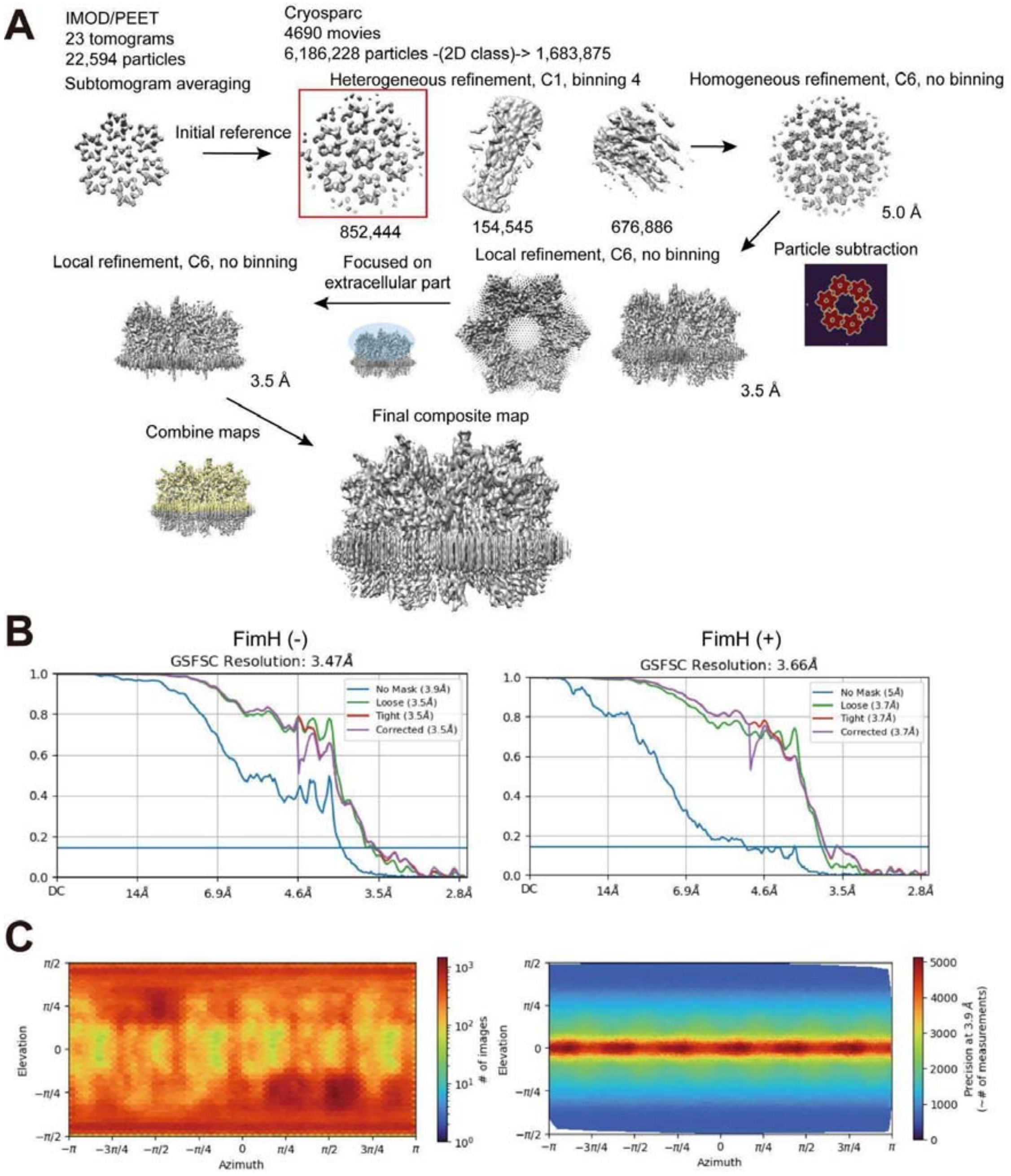

Figure S1

Supplementary Figure 1: Cryo-electron microscopy of the AUM. (A) Summary of the reconstruction procedure. (B) Resolution estimations using Fourier shell correlation. The cut-off value is 0.143. The left and right curves are without and with FimH, respectively. (C) The viewing direction distribution (left) and the posterior precision directional distribution (right). Although the distribution of the views covers nearly entire directions, the precision of the

bioRxiv preprint doi: https://doi.org/10.1101/2023.05.29.542358; this version posted May 29, 2023. The copyright holder for this preprint (which was not certified by peer review) is the author/funder, who has granted bioRxiv a license to display the preprint in perpetuity. It is made available under aCC-BY 4.0 International license.

side views deteriorates due to the overlapping molecules.

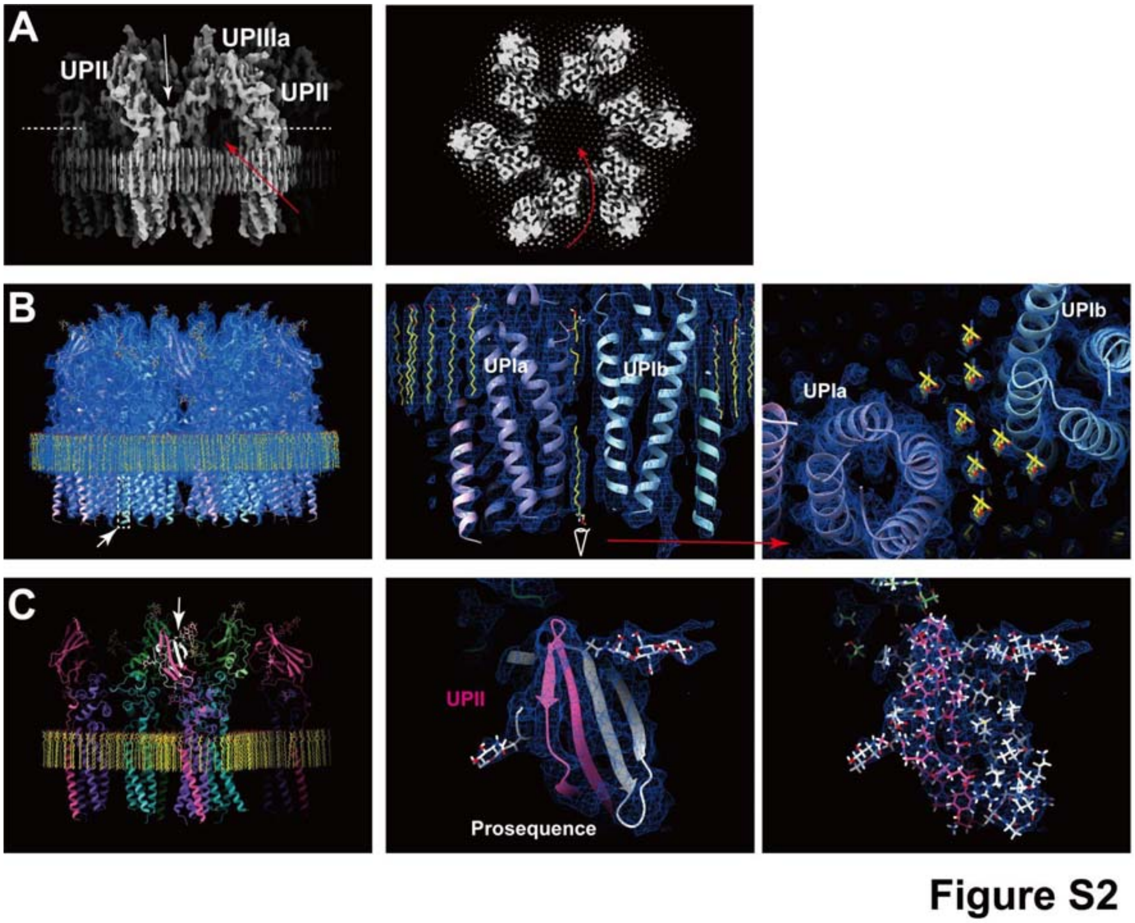

Supplementary Figure 2: Additional structural features. (A) Y-shaped conformation of the uroplakin heterotetramer. The white and red arrows indicate the groove between UPII and UPIIIa, and the channel beneath the II-IIIa arch, respectively. The broken lines indicate the position of the cross-section on the right panal. (B) Weak signals of lipids of the inner leaflet (arrow). The view from the cytoplasmic side (right) shows the hexagonal alignment of the inner lipids (yellow). (C) The prosequence of UPII (arrow, models in white) forms an anti-parallel beta-sheet structure. The electron densities (mesh) validate the assignment of the domain (middle and right).

bioRxiv preprint doi: https://doi.org/10.1101/2023.05.29.542358; this version posted May 29, 2023. The copyright holder for this preprint (which was not certified by peer review) is the author/funder, who has granted bioRxiv a license to display the preprint in perpetuity. It is made available under aCC-BY 4.0 International license.

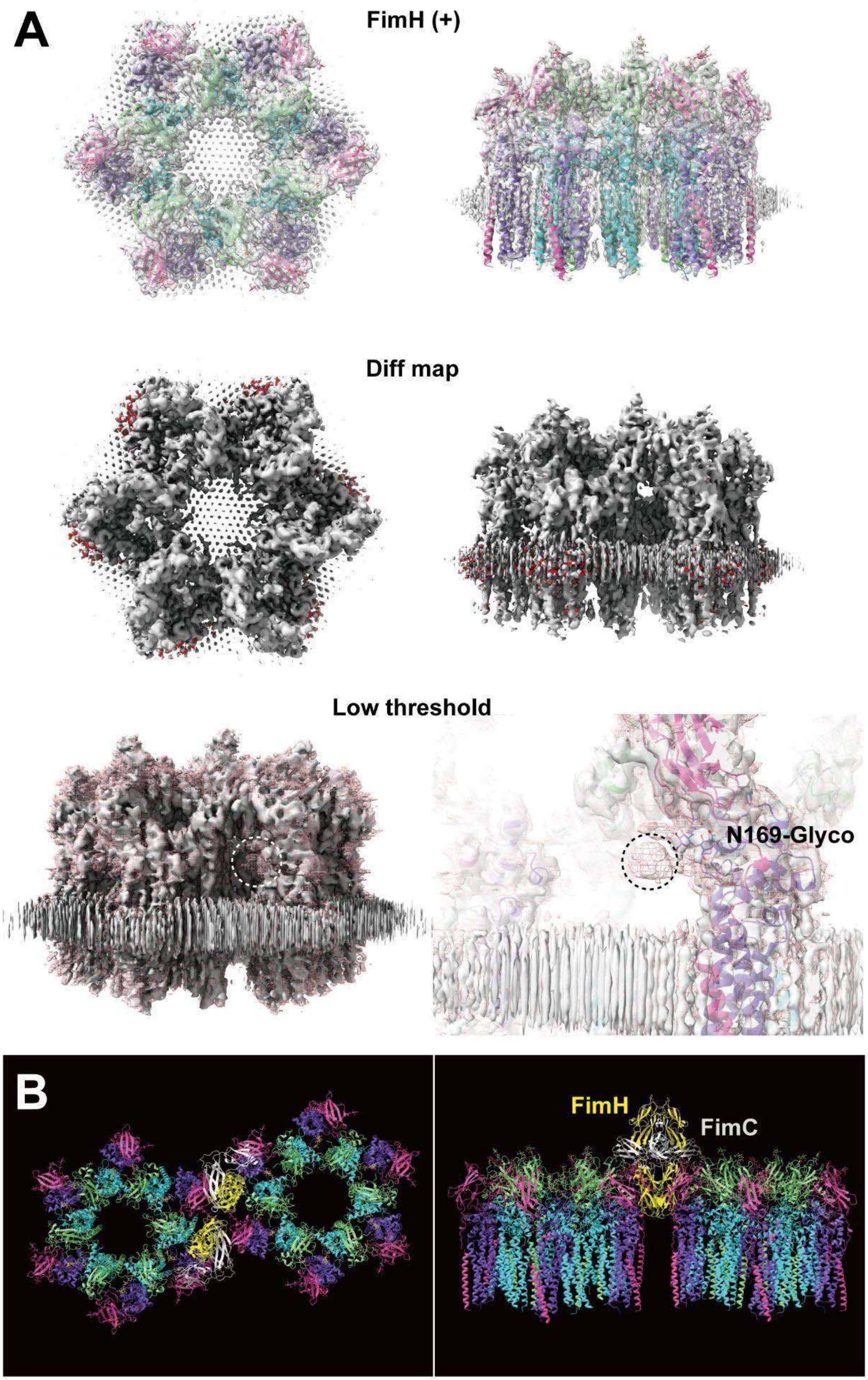

Figure S3

bioRxiv preprint doi: https://doi.org/10.1101/2023.05.29.542358; this version posted May 29, 2023. The copyright holder for this preprint (which was not certified by peer review) is the author/funder, who has granted bioRxiv a license to display the preprint in perpetuity. It is made available under aCC-BY 4.0 International license.

Supplementary Figure 3: FimH-bound uroplakin structure. (A) Uroplakin structure in the presence of FimH-FimC complexes. Model fitting (top) and the difference map (middle) indicate no conformational changes within the uroplakin complex. The difference map shows scattered signals in the lipid regions near the Ia/II heterodimer, suggesting an altered lipid configuration. (B) Model of the uroplakin complex pair with bound FimH-FimC complexes (PDBID: 1QUN). The gap between the two neighboring uroplakin complexes is so narrow that the mannose-binding domain of FimH barely fits.

Supplementary Table 1: Summary of data collection and model validation

**Data collection parameters**	
Magnification	64,000×
Pixel size (Å)	1.35
Voltage (keV)	300
	Tomography
Defocus range (µm)	2.5-4.5
Exposure time (sec/tilt)	0.18
Number of frames per tilt	10
Angular range (°)	±60
Increments (°)	3
Total dose ($e^-/Å^2$)	50
Number of tilt series recorded	95

bioRxiv preprint doi: https://doi.org/10.1101/2023.05.29.542358; this version posted May 29, 2023. The copyright holder for this preprint (which was not certified by peer review) is the author/funder, who has granted bioRxiv a license to display the preprint in perpetuity. It is made available under aCC-BY 4.0 International license.

Number of tilt series processed	23
Initial number of subtomograms	83,109
Final number of subtomograms	22,594
	SPA
Defocus range (µm)	1.1-4.5
Exposure time (sec)	6.7
Number of frames	50
Total dose ($e^-/Å^2$)	50
Tilt angles (°)	0, 30 , 45, 55
Ratio of tilted images	1:1:2:2 for 0, 30, 45, 55°
Number of movies recorded	4,690 (FimH-)/2,733 (FimH+)
Box size (pixels)	512
Initially picked particles	12,764,463 (FimH-)/6,619,664 (FimH+)
Final particles	609,567 (FimH-)/177,303 (FimH+)
**Model validation statistics**	
Initial model used	AlphaFold prediction
Bonds length RSMD (Å)	0.023
Bonds angles RSMD (°)	2.466
MolProbability score	1.74

bioRxiv preprint doi: https://doi.org/10.1101/2023.05.29.542358; this version posted May 29, 2023. The copyright holder for this preprint (which was not certified by peer review) is the author/funder, who has granted bioRxiv a license to display the preprint in perpetuity. It is made available under aCC-BY 4.0 International license.

Clash score	2.55
Rotamer outliers (%)	1.49
Ramachandran plot (%)	Favored: 89.25<br>Allowed: 10.75<br>Outliers: 0.00
CaBLAM outliers (%)	4.37
B-factors (min/max/mean)	59.32/134.09/93.14
Map resolution estimates (Å)	3.5 ($FSC_{half\text{-}map}$=0.143)<br>3.6 (FSC_{model}=0.143)

Supplemental spread sheet 1: Numerical data used for generating boxplot in Figure 7.
